# Partial Mechanical Unloading of the Heart Disrupts L-Type Calcium Channel and Beta-Adrenoceptor Signaling Microdomains

**DOI:** 10.3389/fphys.2018.01302

**Published:** 2018-09-19

**Authors:** Peter T. Wright, Jose L. Sanchez-Alonso, Carla Lucarelli, Anita Alvarez-Laviada, Claire E. Poulet, Sean O. Bello, Giuseppe Faggian, Cesare M. Terracciano, Julia Gorelik

**Affiliations:** ^1^Myocardial Function, National Heart and Lung Institute, Imperial College London, Imperial Centre for Translational and Experimental Medicine, Hammersmith Hospital, London, United Kingdom; ^2^Department of Cardiac Surgery, School of Medicine, University of Verona, Verona, Italy

**Keywords:** cAMP, beta-adrenergic, excitation-contraction, calcium, microdomains, cardiac, t-tubules, unloading

## Abstract

**Introduction:** We investigated the effect of partial mechanical unloading (PMU) of the heart on the physiology of calcium and beta-adrenoceptor-cAMP (βAR-cAMP) microdomains. Previous studies have investigated PMU using a model of heterotopic-heart and lung transplantation (HTHAL). These studies have demonstrated that PMU disrupts the structure of cardiomyocytes and calcium handling. We sought to understand these processes by studying L-Type Calcium Channel (LTCC) activity and sub-type-specific βAR-cAMP signaling within cardiomyocyte membrane microdomains.

**Method:** We utilized an 8-week model of HTHAL, whereby the hearts of syngeneic Lewis rats were transplanted into the abdomens of randomly assigned cage mates. A pronounced atrophy was observed in hearts after HTHAL. Cardiomyocytes were isolated via enzymatic perfusion. We utilized Förster Resonance Energy Transfer (FRET) based cAMP-biosensors and scanning ion conductance microscopy (SICM) based methodologies to study localization of LTCC and βAR-cAMP signaling.

**Results:** β_2_AR-cAMP responses measured by FRET in the cardiomyocyte cytosol were reduced by PMU (loaded 28.51 ± 7.18% vs. unloaded 10.84 ± 3.27% N,n 4/10-13 mean ± SEM ^∗^*p* < 0.05). There was no effect of PMU on β_2_AR-cAMP signaling in RII_Protein Kinase A domains. β_1_AR-cAMP was unaffected by PMU in either microdomain. Consistent with this SICM/FRET analysis demonstrated that β_2_AR-cAMP was specifically reduced in t-tubules (TTs) after PMU (loaded TT 0.721 ± 0.106% vs. loaded crest 0.104 ± 0.062%, unloaded TT 0.112 ± 0.072% vs. unloaded crest 0.219 ± 0.084% N,n 5/6-9 mean ± SEM ^∗∗^*p* < 0.01, ^∗∗∗^*p* < 0.001 vs. loaded TT). By comparison β_1_AR-cAMP responses in either TT or sarcolemmal crests were unaffected by the PMU. LTCC occurrence and open probability (P_o_) were reduced by PMU (loaded TT P_o_ 0.073 ± 0.011% vs. loaded crest P_o_ 0.027 ± 0.006% N,n 5/18-26 mean ± SEM ^∗^*p* < 0.05) (unloaded TT 0.0350 ± 0.003% vs. unloaded crest P_o_ 0.025 N,n 5/20-30 mean ± SEM NS ^#^*p* < 0.05 unloaded vs. loaded TT). We discovered that PMU had reduced the association between Caveolin-3, Junctophilin-2, and Cav1.2.

**Discussion:** PMU suppresses’ β_2_AR-cAMP and LTCC activity. When activated, the signaling of β_2_AR-cAMP and LTCC become more far-reaching after PMU. We suggest that a situation of ‘suppression/decompartmentation’ is elicited by the loss of refined cardiomyocyte structure following PMU. As PMU is a component of modern device therapy for heart failure this study has clinical ramifications and raises important questions for regenerative medicine.

## Introduction

Mechanical load is a key factor in the control of cardiomyocyte structure and subsequently controls cellular physiology (Ibrahim et al., 2013b). The cardiomyocyte is invaginated at regular intervals by transverse (t)-tubules which allow the formation of dyadic couplings between LTCC and the ryanodine receptors of the intracellular sarcoplasmic reticulum. These structures are integrally important to the control of intracellular calcium (Sanchez-Alonso et al., 2016) and secondary messengers such as cAMP (Nikolaev et al., 2010). They contribute to the faithful control of cardiomyocyte excitation contraction-coupling via the careful compartmentation of intracellular signaling processes. The PMU of healthy hearts disrupts some aspects of TT function (Ibrahim et al., 2012a). It is proposed that the transverse-axial tubules system (TATS) exists in dynamic equilibrium whereby both under and overloading scenarios can distort the system (Terracciano et al., 2009; Ibrahim et al., 2010, 2012a). The unloading of previously overloaded failing hearts can normalize cellular structure and subsequently cellular function (Ibrahim et al., 2012b). The potentially pathological effects of PMU are clinically important due to the common deployment of LVADs as therapy for heart failure (Holmberg et al., 2017). Although, LVADs are of crucial importance as a ‘bridge to transplant,’ the explant rate remains low (Drakos et al., 2012). It would therefore be useful to explore the subtleties of ‘ineffective’ unloading of cardiomyocytes with a view to better understanding how to tune therapies to provide the correct regenerative environment.

Previously, Ibrahim et al. (2012a) studied PMU of healthy hearts with a HTHAL model using an 8-week period of unloading. They compared this against control hearts and those of animals which had received trans-aortic constriction (hypertrophy). Isolated cardiomyocytes from healthy hearts which had received PMU were atrophied, had aberrant cytoarchitecture and calcium handling. The morphology of the calcium transient was altered (lower amplitude and longer times to peak and relaxation) in comparison to control. Importantly, like the model of overload, the cells had an increased frequency of calcium sparks and altered spark morphology. These changes were attributed to the loss of normal TATS organization. However, the localized signaling functions of molecules such as the LTCCs and β-adrenergic receptors (βARs) which are fundamental to excitation-contraction processes within the cells, were not investigated in detail in these studies.

We sought to examine the role of unloading in organizing molecular activity at the level of the cell membrane. We employed an animal model of partial myocardial mechanical unloading by performing HTHALs (Ibrahim et al., 2013a). We enzymatically isolated cells from healthy hearts and hearts after 8 weeks of unloading. The cells’ structural parameters were studied with confocal and SICM. The localized signaling of LTCCs was studied with a modified SICM technique which allows the selective patching of specific membrane sub-domains such as TT openings or sarcolemmal crests (Bhargava et al., 2013). βAR function was assessed by monitoring sub-cellular cAMP responses using FRET-based biosensors (Nikolaev et al., 2004; Di Benedetto et al., 2008; Wright et al., 2018). These biosensors were delivered to either the cell cytosol or membrane regions using specific molecular tags. This allowed us to monitor the function of β_1_AR and β_2_AR sub-types within different sub-cellular microdomains. The localization of β_2_AR-cAMP activity was studied using a further modification of SICM, which combines SICM and FRET techniques (Nikolaev et al., 2010). This allows the study of cAMP activity in specific regions of the cell by permitting the acquisition of cellular membrane topography and localized application of agonist via the SICM probe. Any cAMP responses evoked by the local treatment can be measured concurrently using FRET biosensors. Using this technique, we discovered a suppression of β_2_AR-cAMP but not of β_1_AR-cAMP signaling. LTCCs signaling was suppressed by unloading, but this was dynamic and occurred without the removal of specific ‘activatable’ LTCC capacity from the cell.

## Materials and Methods

### Animals and Ethics Statement

Approval for this work was obtained from the Animal Welfare and Ethics Review Board (AWERB) of Imperial College London. This study was carried out in accordance with United Kingdom Home Office guidelines (ASPA 1986 and EU directive 2010/83) and conformed to all protocols of Imperial College London. All procedures were carried out on male, syngeneic, Lewis rats (150–250 g) obtained from Charles River Laboratories (Margate, United Kingdom). Animals were kept at 25°C in ventilated cages and fed standard chow. Animals were randomly assigned to be either donors or recipients. Recipient animals received ‘healthy’ heart and lung grafts from the first group. Following 8 weeks of maintenance, and upon sacrifice, cells isolated from implanted (heterotopic) hearts are defined as **Unloaded** and cells from the animal’s thoracic (orthotopic) heart are defined as age-matched **Loaded** control.

### Heterotopic Heart and Lung Transplant

A more detailed description of this surgery has been published previously (Ibrahim et al., 2013a). Briefly, the recipient animal was prepared (hair removed and skin sterilized) and an incision was made in the abdomen. The mesentery and bowel were carefully removed and wrapped in damp, sterile swabbing material. The IVC and aorta were revealed and undermined. They were manipulated with 2-0 suture and clamped. A 2–3 mm incision was made in the aorta. The heart and lung block was removed from storage solution and carefully wrapped in swab, soaked in cold cardioplegia, which was refreshed periodically throughout the operation. The tissue block was maneuvered toward the abdominal cavity, an 8-0 prolene suture was passed through the aorta at the 5 o’clock position outside-in. The suture was then passed through the aortic incision at the 7 o’clock position – the anastomosis then continued clockwise–counter-clockwise, respectively, until it was sealed at the 8 o’clock position on the aorta with three surgeons’ knots. Following careful removal of the swabbing protecting the heart and orientation of the tissue, the clamps were removed to allow the perfusion of the heart, as well as the restoration of circulation to the limbs distal to the anastomosis. Hemostasis was provided around the anastomosis site until bleeding ceased. The beating rate and strength of contraction of the implanted heart was assessed. Once the graft activity was deemed optimal the gut was returned and carefully orientated within the abdominal cavity. The abdominal musculature was closed with 5-0 vicryl suture in a continuous pattern. The animal’s skin was closed with 4-0 vicryl sutures using a continuous sub-cuticular suture pattern. Carprofen was provided (1.25 mg, s.c.) for analgesia and enrofloxacin (2.5 mg, s.c.) as an antibiotic. Fluid was also given (2 ml saline s.c.). Animals were allowed to recover for about 45 min in a heated chamber, before being transferred individually to standard cages. Operated animals received carprofen and enrofloxacin s.c at 24 and 48 h post-operation and soft food. They were returned to normal chow after ∼72 h.

### Unloaded Heart Harvest

After 8 weeks the recipient animals were anesthetized with isoflurane. The abdomen was incised and bowel manipulated to reveal the implanted heart. The aorta and IVC were manipulated and clamped. The heart and lung block were extracted and placed in ice cold Krebs solution. The diaphragm and thorax were incised and opened to allow the removal of the loaded heart. The heart and lung block were excised and placed in ice-cold Krebs.

### Cell Isolation

Both loaded and unloaded hearts were retrogradely perfused with Krebs-Henseleit solution (37°C, 95% O_2_, 5% CO_2_ 1 mM Ca^2+^) using a Langendorff perfusion apparatus. After beating was properly established the heart was arrested by perfusion with a low calcium buffer for 5 min. The heart was then perfused with a mixture of collagenase:hyaluronidase (1 mg/ml:0.6 mg/ml) for 10 min. After this period, the heart was cut down, the atria and right ventricle were removed. The ventricle was then disrupted and the mixture shaken mechanically in collagenase/hyaluronidase solution (35°C). After 30 min this yielded single isolated cardiomyocytes. These mixtures were centrifuged 1,000 rpm 2 min. Cell pellets were re-suspended in buffer not containing enzymes.

### Förster Resonance Energy Transfer (FRET) Microscopy

Isolated cardiomyocytes were centrifuged, re-suspended in M199 and around 5,000 plated on individual laminin coated 25 mm coverslips. After attachment they were washed with new M199 and cultured for 48 h in M199 in the presence of adenovirus encoding the RII_Epac or cEPAC2 cAMP sensors (Di Benedetto et al., 2008; Nikolaev et al., 2010; MacDougall et al., 2012). FRET experiments were conducted with ORCA-4.0 CCD camera’s using DualView or QuadView beamsplitters. Both FRET construct consist of CFP and YFP complexed with an exchange protein activated by cAMP (epac)-derived cAMP binding domain. In the case of the RII_Epac sensor the construct also contains the regulatory II subunit of PKA, as a result this sensor aligns to the cardiomyocyte sarcomere in conjunction with A-kinase anchoring proteins. Binding of cAMP to this sensor indicates the penetration of cAMP into PKA domains, whereas cEPAC2 indicates cytosolic responses. After baseline FRET measurements in the presence of 100 nM CGP20712A (to block β_1_AR) or 50 nM ICI118, 551 (to block β_2_AR) cells were perfused with 100 nM isoprenaline to stimulate β_1_AR or β_2_AR. Following this phase NKH477 (5 μM) was perfused onto the cells to fully stimulate adenylate cyclase and allow the correction of β_2_AR-cAMP responses to maximal cAMP responses.

### Scanning Ion Conductance Microscopy

Scanning ion conductance microscopy is a scanning probe microscopy technique based upon the ability to reconstruct topographical images of surfaces on the basis of changes in conductance at the tip of a nanopipette. The physical basis of this technique has been explained in exhaustive detail previously (Novak et al., 2009). Briefly, the sample in this case the cell, is rastered underneath a scanning pipette and a high-resolution image of the cell is constructed. Once this image has been acquired the tip of the pipette can be localized to very specific positions on the cell surface.

### Super Resolution (Smart) Patch Clamp

This technique has been explained in detail elsewhere. Following the acquisition of a topographical image of the cell with SICM an area of interest is selected. The pipette is moved to a neutral area of the dish and clipped to give the patch-clamp resistance. It is then moved back to the original area of interest and the pipette is fused with the membrane patch and calcium channel recordings made as described in the references above. A sub-population of cells were treated with the non-specific calcium channel activator BAYK8644 (Tocris Biosciences, United States).

### SICM/FRET

This technique has also been employed and described in more detail elsewhere. Following the acquisition of cell topography, as TT or sarcolemmal crest region was targeted and ISO was applied via electrophoresis. FRET response is measured as described above (Nikolaev et al., 2010; Wright et al., 2014).

### Whole-Cell Patch Clamp Recordings

L-type calcium currents (ICa, L) were recorded using the whole-cell patch-clamp configuration with the external recording solution of the following composition (in mmol/L): 140 NaCl, 6 KCl, 10 glucose, 10 HEPES, 1.5 MgCl_2_, 1 CaCl_2_, pH 7.4 with 1M NaOH. An internal pipette solution contained (in mmol/L): 100 Cs-methanesulfonate, 40 CsCl, 10 HEPES, 5 EGTA, 5 Mg-ATP free acid, 0.75 MgCl_2_, pH 7.2 with CsOH. Patch pipettes had mean resistances of 3.5–5 MΩ. Currents were recorded using an Axopatch-1D amplifier connected to a Digidata 1322A acquisition system (Axon Instruments, Foster City, CA, USA). The bath was connected to the ground via a reference electrode containing Ag–AgCl pellet. Data were low-pass filtered at 2 kHz using the Bessel filter of the amplifier and sampled at 2 kHz. All recordings were performed at room temperature (22–24°C). ICa, L channel activity was recorded during 200 ms from a holding potential of -40 mV to test potentials ranging from -40 to +60 mV, with pulses applied every 2 s in 5 mV increments. Current amplitude at 0 mV was taken as a peak current and divided by a capacitance value for each cell (current density, pA/pF).

### Measurement of Cav3, T-Cap and Junctophilin-2 Density and Regularity and Proximity Ligation Assay

Isolated cells were plated on 13 mm coverslips, fixed with methanol (-20°C) and kept in PBS at 4°C. Proximity ligation assay was performed with the Duolink (inSitu) kit (Sigma-Aldrich, United Kingdom) using anti-Goat, anti-Rabbit or anti-Mouse reagents as required with Duolink ‘green’ detection reagents (Sigma-Aldrich, United Kingdom), as per the manufacturers instructions. Primary antibodies for JPH-2 (Goat polyclonal, Santa Cruz, sc - 51313), caveolin-3 (Cav3, mouse monoclonal 610421) and Cav1.2 (Alomone, Rabbit polyclonal). Cell imaging was performed with 60x oil immersion Zeiss lens, using inverted confocal laser scanning microscope (A Zeiss LSM-780), with argon laser beam at specific wavelengths for the secondary antibodies (Alexa Fluor Cav3 546 nm). Protein density and respective regularity of distribution were obtained with the same method used for TTs analysis described in more detail elsewhere (Wright et al., 2018). PLA analysis was performed by automatically thresholding cell images in ImageJ (Image J Corp, United States) and utilizing default plugins to measure area covered by particles, size of particles, number of particles and number of particles per μm.

### di-8ANNEPPS Staining of Freshly Isolated Cardiomyocytes

After staining freshly isolated cells with di-8ANNEPPS image stacks were obtained with a Zeiss LSM710 confocal microscope as previously described (Schobesberger et al., 2017). A 40 × 5 μm area was selected and binarized. Density and power of regularity were analyzed as per our laboratories protocols. Density was defined as the percentage of black pixels. Power of regularity was calculated using a bespoke MATLAB code by plotting a waveform of the binarized image of the cell and the Fast-Fourier transform was used to plot a representative curve of the frequency of di8ANNEPPS stained ultrastructure. The strength of the peak at ∼0.5 micron indicated the regularity of di8ANEPPS staining and therefore TT structure.

### Statistical Analysis

Where unrelated two populations were analyzed statistical difference was determined using an unpaired (two-tailed) Students’ *T*-test. Where more than two unrelated populations were analyzed, a one-way ANOVA with a Bonferroni post-test (*post hoc*) was used. This was performed by using Graph-Pad 4.0 software. In the figure legends, N refers to the number of preparations, *n* refers to the number of measurements.

## Results

### Partial Mechanical Unloading Differentially Distorts βAR-cAMP Microdomains

**FIGURE 1:**
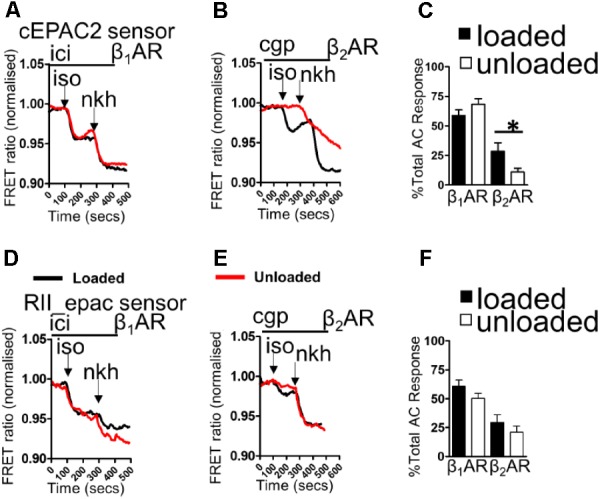
**(A)** Illustrative traces showing β_1_AR-cAMP (ICI+ISO) and full AC-cAMP (NKH) responses measured by FRET microscopy using the cEPAC2 sensor in loaded and unloaded cells. **(B)** Illustrative traces showing β_2_AR-cAMP (CGP+ISO) and full AC-cAMP (NKH) responses measured by FRET microscopy using the cEPAC2 sensor in loaded and unloaded cells. **(C)** Graph presenting mean β_1_AR-cAMP and β_2_AR-cAMP responses as a function of total AC responses for loaded and unloaded cells. cEPAC2 sensor, β_1_AR-cAMP (loaded 58.78 ± 4.82% vs. unloaded 68.12 ± 4.85% N,n 5/10-13 mean ± SEM, NS). cEPAC2, β_2_AR-cAMP (loaded 28.51 ± 7.18% vs. unloaded 10.84 ± 3.27% N,n 4/10-13 mean ± SEM ^∗^*p* < 0.05). **(D)** Illustrative traces showing β_1_AR-cAMP (ICI+ISO) and full AC-cAMP (NKH) responses measured by FRET microscopy using the RII_epac sensor in loaded and unloaded cells. **(E)** Illustrative traces showing β_2_AR-cAMP (CGP+ISO) and full AC-cAMP (NKH) responses measured by FRET microscopy using the RII_epac sensor in loaded and unloaded cells. **(F)** Graph presenting mean β_1_AR-cAMP and β_2_AR-cAMP responses as a function of total AC responses for loaded and unloaded cells. RII_epac sensor, β_1_AR-cAMP (loaded 60.64 ± 5.45 vs. unloaded 50.21 ± 4.44 N,n 4/5-6 mean ± SEM, NS). RII_epac, β_2_AR-cAMP (loaded 29.47 ± 6.55% vs. unloaded 21.02 ± 5.14% N,n 4/6-8 mean ± SEM NS).

Loaded and unloaded cells were transfected with cEPAC2 or RII_epac sensors and cultured for 48 h. β_1_AR-cAMP and β_2_AR-cAMP were then assessed as a function of the total cAMP response. Unloading did not affect the β_1_AR-cAMP response or the overall cellular cAMP response in the cell cytosol (**Figures 1A,C**). Unloading reduced the β_2_AR-cAMP responses within the cell cytosol (**Figures 1B,C**). Withdrawal of mechanical load reduced the amount of cAMP penetrating cellular RII_PKA domains but this difference did not reach statistical significance (**Figures 1D,F**). No difference was observed in total RII domain cAMP responses. Specific stimulation of β_2_AR-cAMP elicited a slightly higher response in RII_PKA domains, in control cells. However, similar to experiments studying β_1_AR-cAMP this effect was also not statistically significant (**Figures 1E,F**). In summary, unloading only significantly reduced the cytosolic β_2_AR-cAMP response.

### Partial Mechanical Unloading Selectively Reduces β_2_AR-cAMP Microdomain Responses

**FIGURE 2:**
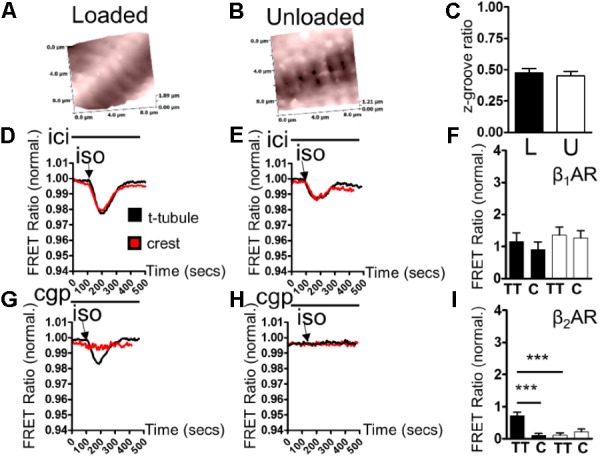
**(A)** Illustrative topographical image of a loaded control cardiomyocyte membrane acquired with SICM. **(B)** Illustrative topographical image of an unloaded control cardiomyocyte membrane acquired with SICM. **(C)** Graph comparing the z-groove ratios of loaded and unloaded cells (loaded 0.473 ± 0.035 vs. unloaded 0.451 ± 0.035 N,n 5/(36-40) mean ± SEM NS). **(D)** Illustrative traces showing β_1_AR-cAMP (ICI+ISO) responses elicited in t-tubule or crest regions by combination SICM/FRET microscopy using the cEPAC2 sensor in a loaded cell. **(E)** Illustrative traces showing β_1_AR-cAMP (ICI+ISO) responses elicited in t-tubule or crest regions by combination SICM/FRET microscopy using the cEPAC2 sensor in an unloaded cell. **(F)** Graph comparing β_1_AR-cAMP responses evoked in t-tubule (TT) or crest (C) regions by SICM/FRET in loaded and unloaded cells (loaded t-tubule 1.155 ± 0.27% vs. loaded crest 0.903 ± 0.24%, unloaded t-tubule 1.356 ± 0.25% vs. unloaded crest 1.259 ± 0.232% N,n 5/6-9 mean ± SEM NS). **(G)** Illustrative traces showing β_2_AR-cAMP (CGP+ISO) responses elicited in t-tubule or crest regions by combination SICM/FRET microscopy using the cEPAC2 sensor in a loaded cell. **(H)** Illustrative traces showing β_2_AR-cAMP (CGP+ISO) responses elicited in t-tubule or crest regions by combination SICM/FRET microscopy using the cEPAC2 sensor in an unloaded cell. **(I)** Graph comparing β_2_AR-cAMP responses evoked in t-tubule (TT) or crest (C) regions by SICM/FRET in loaded and unloaded cells (loaded t-tubule 0.721 ± 0.106% vs. loaded crest 0.104 ± 0.062%, unloaded t-tubule 0.112 ± 0.072% vs. unloaded crest 0.219 ± 0.084% N,n 5/6-9 mean ± SEM ^∗∗^*p* < 0.01, ^∗∗∗^*p* < 0.001 vs. loaded t-tubule).

Scanning ion conductance microscopy scans showed similar degrees of sarcolemmal organization in loaded and unloaded cardiomyocytes (**Figures 2A,B**) (freshly isolated in preparation for LTCC experiments). Z-groove ratios were calculated for multiple scans and the relative degrees of cell membrane organization were analyzed. Mean Z-groove ratios were no different for the loaded and unloaded cell groups (**Figure 2C**). As a result it can be reported that PMU does not produce its effect on cytosolic β_2_AR-cAMP responses by reducing the organization of the cellular sarcolemma. In control, loaded cells (cultured for 48 h) β_1_AR-cAMP responses were elicited in both tubule and crest domains after application of ISO in the presence of ICI (β_2_AR blockade). This is consistent with previous studies of this particular sensor (**Figure 2D**). Unloading did not appear to alter β_1_AR-cAMP responses in tubule or crest domains (**Figure 2E**). Analysis revealed no statistical difference between β_1_AR-cAMP responses from the TT or crest regions of loaded or unloaded cardiomyocytes (**Figure 2F**). Consistent with previous work, application of ISO in the presence of CGP (β_1_AR blockade) only elicited β_2_AR-cAMP responses on t-tubular surfaces (**Figure 2G**). Mechanical unloading significantly reduced the β_2_AR-cAMP response on cellular t-tubular structures (**Figures 2H,I**), which is consistent with the effect of unloading on the ‘whole-cell’ stimulation of β_2_AR-cAMP responses measured with the cEPAC2 sensor. Cell crests yielded very little β_2_AR-cAMP response in the control condition and the unloaded cells were no different (**Figures 2G,I**).

### Partial Mechanical Unloading ‘Silences’ L-Type Calcium Channel (LTCC) Activity

**FIGURE 3:**
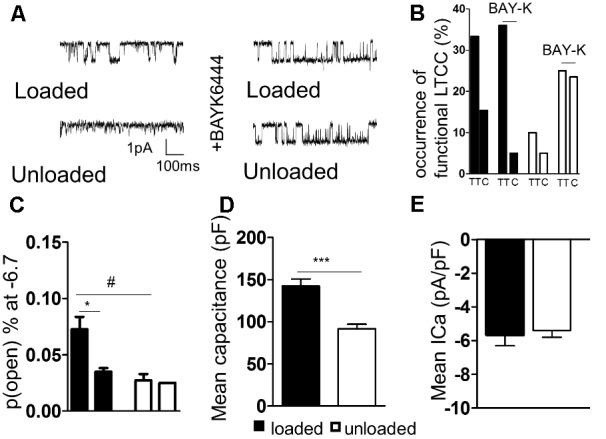
**(A)** Illustrative traces of LTCC activity recorded in ‘cell-attached’ mode from loaded and unloaded cells. **(B)** Graph comparing the occurrence functional LTCC’s within different cellular microdomains of loaded or unloaded cells. A sub-population of loaded and unloaded cells were treated with the non-specific LTCC agonist BAYK8644. **(C)** Graph comparing the open probability of LTCC’s within different cellular microdomains of loaded or unloaded cells (loaded t-tubule 0.073 ± 0.011% vs. loaded crest 0.027 ± 0.006% N,n 5/18-26 mean ± SEM ^∗^*p* < 0.05) (unloaded t-tubule 0.0350 ± 0.003% vs. unloaded crest 0.025 N,n 5/20-30 mean ± SEM NS). ^#^*p* < 0.05 vs. loaded t-tubule. **(D)** Graph comparing the mean capacitance of loaded and unloaded cardiomyocytes (loaded 142.1 ± 8.65 pF vs. unloaded 91.67 ± 5.40 pF N,n 5/11-15 mean ± SEM ^∗∗∗^*p* < 0.001). **(E)** Graph comparing the mean I_Ca_ of loaded and unloaded cardiomyocytes (loaded -5.67 ± 0.64 pA/pF vs. unloaded –4.62 ± 0.46 pA/pF N,n 5/11-14 mean ± SEM NS).

TT LTCC activity was faithfully recorded from freshly isolated loaded cardiomyocytes (**Figure 3A**). In comparison, LTCC activity was suppressed in unloaded cardiomyocytes (**Figure 3A**). The occurrence of LTCC channels, the number of channels recorded vs. successful patches, was higher in TT membranes of loaded cells than cell crests (**Figure 3B** and **Supplementary Figure S1A**). In comparison a far lower occurrence of LTCCs was observed in the TT membrane and cell crests of unloaded cells. The difference between TT and crest domains observed in loaded controls was lost after unloading. The specific LTCC channel activator BAY-K was employed to assess whether the channels had been lost or whether their activity was being suppressed. LTCC activity was recorded in TT domains in both conditions (**Figure 3A**). BAY-K treated loaded cells showed the same occurrence of LTCCs as observed in untreated cells. In comparison BAY-K treatment revealed a large amount of LTCCs in both the TT and crest domains of unloaded cells (**Figure 3B**). The suggestion is that LTCCs are silenced by unloading and that this treatment removes the restriction of the channels to TT domains. The biophysical characteristics of LTCCs are also altered by unloading. In loaded cells the probability of a calcium channel being open is greater in the TT domain than in the crest (**Figure 3C** and **Supplementary Figure S1B**). In unloaded cells the open probability is also decreased, alongside the occurrence. To assess whether these alterations were as a result of gross changes in calcium handling, we assessed ‘whole-cell’ calcium current. We discovered that unloaded cells had a lower capacitance in comparison to loaded cells, which is due to their smaller size (**Figure 3D**). Whole-cell calcium current was no different between loaded and unloaded cells following correction for capacitance (**Figure 3E**). This suggests that differences in LTCC function are directly attributable to the activity of the channels within the membrane microdomain.

### Partial Mechanical Unloading Alters the Association of Structural Proteins Crucial for LTCC Function

**FIGURE 4:**
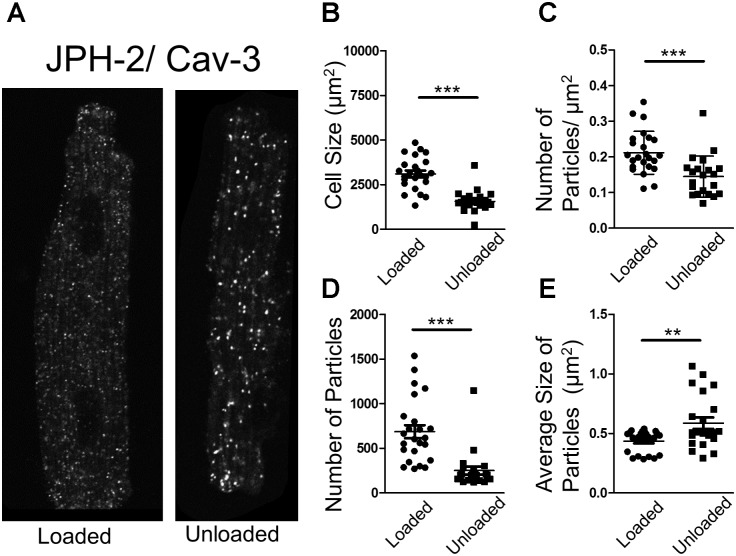
**(A)** PLA image demonstrating the association of JPH-2 and Cav-3 in loaded and unloaded cells. **(B)** Graph comparing the size of loaded and unloaded cells (loaded 3104 ± 186.1 μm^2^ vs. unloaded 1561 ± 128.9 μm^2^ N,n 3/22-24 mean ± SEM ^∗∗∗^*p* < 0.001). **(C)** Graph comparing the number of particles/μm^2^ in loaded and unloaded cells (loaded 0.212 ± 0.012 particles/μm^2^ vs. 0.145 ± 0.013 particles/μm^2^ N,n 3/21-24 mean ± SEM ^∗∗∗^*p* < 0.001). **(D)** Graph comparing the number of particles in loaded and unloaded cells (loaded 685.9 ± 73.33 vs. unloaded 250.3 ± 46.01 N,n 3/21-24 mean ± SEM ^∗∗∗^*p* < 0.001). **(E)** Graph comparing the average size of particles in loaded and unloaded cells (loaded 0.435 ± 0.019 μm^2^ vs. unloaded 0.587 ± 0.048 μm^2^ N,n 3/21-24 mean ± SEM ^∗∗^*p* < 0.01).

**FIGURE 5:**
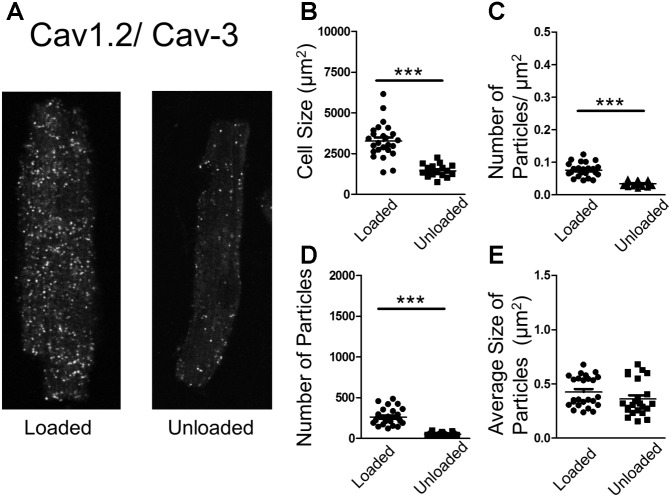
**(A)** PLA image demonstrating the association of Cav1.2 and Cav-3 in loaded and unloaded cells. **(B)** Graph comparing the size of loaded and unloaded cells (loaded 3271 ± 212.4 μm^2^ vs. unloaded 1444 ± 92.03 μm^2^ N,n 3/17-25 mean ± SEM ^∗∗∗^*p* < 0.001). **(C)** Graph comparing the number of particles/μm^2^ in loaded and unloaded cells (loaded 0.076 ± 0.0042 particles/μm^2^ vs. unloaded 0.034 ± 0.0016 particles/μm^2^ N,n 3/23-25 mean ± SEM ^∗∗∗^*p* < 0.001). **(D)** Graph comparing the number of particles in loaded and unloaded cells (loaded 261.9 ± 20.61 vs. unloaded 50.52 ± 3.70 N,n 3/23-25 mean ± SEM ^∗∗∗^*p* < 0.001). **(E)** Graph comparing the average size of particles in loaded and unloaded cells (loaded 0.425 ± 0.028 μm^2^ vs. unloaded 0.361 ± 0.03 μm^2^ 3/23-25 N,n mean ± SEM NS).

In an attempt to understand the suppression of LTCC activity, we performed proximity ligation assays (PLA) on loaded and unloaded cells, which were isolated and fixed. PLA quantifies the association of peptides by measuring fluorescence produced by antibody constructs using confocal microscopy. The size and number of fluorescent particles were assessed. We utilized this method to explore the association of JPH-2 and Cav-3, two proteins crucial for the control of sub-cellular LTCC signaling microdomains. We also assessed the association between Cav-3 and the Ca_v_1.2 sub-unit of the LTCC itself. Unloading reduced the size of cardiomyocytes and reduced the density and regularity of Cav-3 staining (**Supplementary Figures S2A–D**). The staining of cells with di-8-ANNEPPS also reveals the effect of PMU on cell area and aspect ratio, as well as cellular ultrastructure. PMU causes the loss of TT regularity but not TT density (**Supplementary Figures S3A–F**). The JPH-2/Cav-3 PLA demonstrated that the cell size was reduced by unloading (**Figures 4A,B**). The number of particles per micron and total number of particles were decreased by the partial withdrawal of mechanical load (**Figures 4C,D**). This suggested that after unloading the degree of association between these two structural proteins was lower and less organized. Interestingly, the size of particles increased suggesting that where these associations occur there is an agglomeration of the peptides (**Figure 4E**). The Ca_v_1.2/Cav-3 PLA demonstrated once again that cell size was reduced by unloading (**Figures 5A,B**). The number of particles by area and total number was also decreased following unloading (**Figures 5C,D**). Unlike the JPH-2/Cav-3 PLA there was no increase in particle size (**Figure 5E**). This suggested that the association between Ca_v_1.2/Cav-3 was simply reduced following unloading.

## Discussion

Partial mechanical unloading profoundly reduces cardiomyocyte size and modulates the structure and organization of TATS (Ibrahim et al., 2010, 2012a). It has been demonstrated to increase the frequency and persistence of calcium ‘sparks.’ Spark activity is speculated to be elicited by ryanodine receptors and the loss of organization of the sarcomeric dyad following the withdrawal of load. We sought to assess the effect of PMU on the physiology of LTCC’s and βARs. The physiology of βAR-cAMP was altered in a sub-type and microdomain specific way. PMU did not reduce the amplitude of β_1_AR-cAMP responses elicited by whole-cell perfusion and measured in the cell cytosol or RII-PKA domains. PMU reduced the amplitude of β_2_AR-cAMP responses in the cytosol but not in the RII-PKA domains. This is an intriguing finding, as previous papers (MacDougall et al., 2012; Wright et al., 2018) which have employed these two sensors have demonstrated that β_2_AR is a compartmentalized receptor in healthy cardiomyocytes, which will produce cAMP responses in the cell cytosol but not in the RII_PKA domains. The data we present suggest that the production of β_2_AR-cAMP is load-sensitive and may involve the loss of receptor subunits. In the situation of PMU-induced atrophy this is potentially superimposed on a cell which has lost structural integrity via the loss of important components of the cAMP compartmentation machinery such as TTs. As a result we are not observing the increase of absolute β_2_AR-cAMP capacity, in fact we witness its reduction, and this is concomitant with increased persistence and subsequent enhancement of its efficacy due to the PMU-induced loss of cAMP compartmentation. We only employed a saturating level of stimulation for β_1_AR but prior reports (Agarwal et al., 2011) have demonstrated that at sub-maximal levels β_1_AR may act in a compartmentalized fashion. As a result, the effects of PMU may also affect β_1_AR-cAMP in different scenarios. We posit that the primary factor in the lack of an effect of PMU on β_1_AR activity may be a combination of its relative lack of ‘compartmentation’ by important subcellular structures such as TTs. Therefore their loss after PMU has less of an effect on this receptor. Equally, the supra-maximal stimulation afforded by our selected dose of isoprenaline may mean the effects of PMU on β_1_AR-cAMP have eluded us in this case.

The effects of PMU on membrane microdomains were investigated by stimulating β_1_AR or β_2_AR-cAMP responses via local application of agonist and measuring responses evoked in the cardiomyocyte cytosol. β_1_AR-cAMP responses in t-tubular or sarcolemmal crest regions were unaffected by PMU. This is concordant with our findings using whole-cell perfusion of agonist. β_2_AR-cAMP responses were of greater amplitude in t-tubular regions than on cardiomyocyte crests. This is comparable with previous reports (Nikolaev et al., 2010; Wright et al., 2014). PMU appeared to suppress the β_2_AR-cAMP responses in t-tubular domains. β_2_AR-cAMP responses in crest regions were unaltered, but were of such a low amplitude in control cells that an effect could not be seen. This is consistent with our measurements made after perfusing the whole cell with agonist. This may suggest that the effect in whole-cell measurements is purely due to the loss of t-tubular β_2_AR-cAMP responses. The effects of PMU on β_2_AR-cAMP responses occurred without an obvious loss of total cAMP production capacity within the cells. Mechanical load could therefore be an essential stimulus for the production of organized t-tubular networks which are capable of conducting normal compartmentalized β_2_AR-cAMP responses. Mechanical load may be a prerequisite to allow the cells to produce β_2_AR-cAMP responses which are faithfully produced in the TT and restricted from the RII_PKA domains. This phenomena would appear to be less significant for β_1_AR-cAMP responses. However, as suggested the relatively liberal control of this receptor may belie more significant effects at sub-maximal levels. The effects of the singular load-dependent ‘suppression/decompartmentation’ behavior observed for β_2_AR-cAMP responses are unclear for the β_1_AR.

Like β_2_AR-cAMP activity, LTCC seem to be confined to t-tubular microdomains. Greater numbers of LTCC were observed in TTs in normally loaded cardiomyocytes. These channels had a higher open probability, suggesting a larger more active population of LTCC in the loaded cardiomyocyte tubule in comparison to crest domains. This is consistent with our previous work (Sanchez-Alonso et al., 2016). Interestingly, following PMU, LTCC seemed to almost disappear from the TTs of cardiomyocytes. LTCC activity was also significantly reduced. This was demonstrated to be due to a ‘silencing’ of LTCC following PMU, as well as the loss of confinement of LTCC to the TT domains. Experiments performed in the presence of BAYK show the silencing effect of PMU. Simply adding this agent activated LTCCs in both TTs and crest domains. This once again raises the suggestion that the activation and maintenance of compartmentation pathways of molecular signaling processes within cardiomyocyte microdomains are load-dependent.

We observed that the organization and association of proteins which are usually involved in the compartmentation of calcium and cAMP in cardiomyocytes was lost following PMU (Chen et al., 2013; Wong et al., 2013). We also specifically observed the agglomeration of JPH-2 and Cav-3 following PMU, alongside the loss of normal association of Ca_v_1.2 subunit of LTCC with Cav-3. JPH-2 has been reported to be essential for the normal formation of TTs (Chen et al., 2013), which in turn control both calcium handling and cAMP. Caveolae are the site of a sub-population of LTCC (Chen et al., 2013). LTCC activation is achieved by the selective phosphorylation of the channel by PKA or CaMKII, depending on the specific compartment involved (Sanchez-Alonso et al., 2016). We also observe the loss of regularity within the TAT network following PMU which is concordant with previous work in our laboratory (Ibrahim et al., 2012a). These findings suggest that mechanical load is responsible for the collection, distribution and organization of the cell membrane and structural proteins which are then able to form and arrange sub-cellular signaling microdomains. This is apparently achieved by the association and sequestration of receptors, channels and other signaling molecules. Loss of this stimulus as well as causing the atrophy of the cell, causes the loss of faithful signaling organization. This could potentially be a passive process.

**FIGURE 6:**
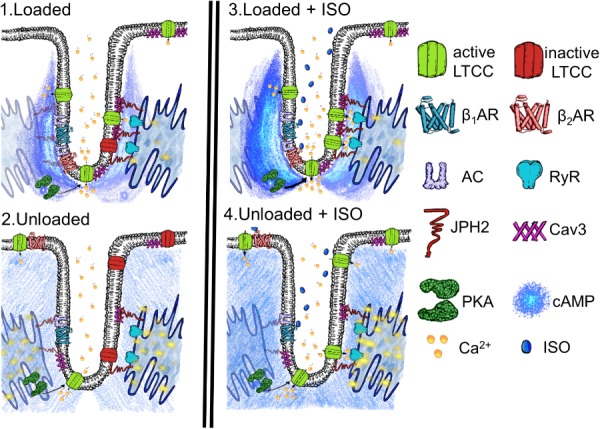
Schematic demonstrating the speculated state of a t-tubular microdomain in loaded or unloaded scenarios with or without isoprenaline stimulation. (1) Loaded cell. LTCC are generally active within the tubule owning to some constitutive cAMP production and consequent PKA activity, scaffolded by Cav-3 and JPH-2. Calcium spark activity at the sarcoplasmic reticulum is nominal. (2) Unloaded cell. LTCC are generally inactive as there is a loss of coherent confinement of cAMP and PKA activity to the t-tubular microdomain. This is due to the mechanosensitivity of the association between Cav1.2, Cav-3, and JPH-2 and the loss of t-tubules. Even though there is a smaller amount of LTCC activity spark activity increases as orphaned RyR no longer receive effective control and thus experience a gain of function. (3) Loaded cell stimulated with ISO. After stimulation β2AR increase cAMP levels in the cytoplasm of the sarcomeric dyad and this activates a sub-population of PKA which activates almost all LTCC. Spark activity remains nominal as effective measures to control LTCC and RYR phosphorylation and the degradation of cAMP remain effective due to scaffolding by the cellular microdomain. (4) Unloaded cell stimulated with ISO. Despite a reduction in the amount of cAMP response initiated by β_2_AR in the cytosol, signals still reach RII_PKA domains, due to the loss of efficient cAMP compartmentation. This stimulates further aberrant spark activity due to the relatively uncontrolled calcium influx in the cell and EC mediator hyperphosphorylation following the loss of normal structure within the sarcomeric dyad following unloading.

Hypertrophy is stimulated by the increased demands placed upon the myocardial tissue, so-called overload, which causes two separate phases of cell behavior. The myocardium exhibits marked structural and functional plasticity, this property is largely due to the modulation of individual cardiomyocytes (Frisk et al., 2016; Watson et al., 2016). Initially, the cell grows and copes with the increased demand and ‘compensates.’ This is followed by an energetic crisis where the biomechanical properties of the cardiomyocytes are stretched to a point beyond which neither adequate functional capacity or maintenance can be sustained. This is referred to as decompensation and leads to myocardial failure. During these phases, the TATS is progressively altered. Atrophy most likely represents the converse of hypertrophy. It has been reported that the integrity of the TAT network is disrupted by the degree and timescales of mechanical unloading (Ibrahim et al., 2012a). As a result it can be posited that as the demand for myocardial function is withdrawn by unloading, the cardiomyocytes adapt by reducing their structural and organizational complexity, which are energetically expensive to sustain. The result is a passive rather than active loss of cardiac structure. This nonetheless results in a pathological loss of cardiac fitness and capacity. We suggest that unloading causes a situation like the one presented in our schematic in **Figure 6**. We suggest that the loss of TT organization removes the innate activity of molecules such as LTCC. The disorganization of TTs and structural proteins seems to result in the increased malignancy of calcium spark activity, as previously reported, (Ibrahim et al., 2012a) despite this loss of LTCC activity. We suggest this may be the effect of the loss of coherent molecular organization. It also reduces the capacity of β_2_AR to signal once stimulated with isoprenaline, the necessity of effective localization of the molecules to produce normative function suggests this effect is in someways similar to the effect on LTCC. Finally, the loss of normal activity in the cytosol but continuing stimulation of RII_PKA domains by β_2_AR after activation is further indicative of a loss of molecular control leading to a scenario in which one receives ‘more bang for the buck’ after excitation due to mechanosensitive decompartmentation.

The role of βAR in the pathophysiology is well characterized but somewhat poorly understood. It is known that chronic βAR stimulation is cardiotoxic and pro-arrhythmic (Engelhardt et al., 1999; Nguyen et al., 2015; Lucia et al., 2018). In settings of heart failure the sympathetic nervous system is upregulated in an effort to produce greater cardiac inotropy, the long term effects of this as described above are deleterious and this gives rise the paradoxical efficacy of ‘beta-blockers’ in maintaining patients with cardiac pathology (Baker et al., 2011). This aspect presents an equally complex picture as β_1_AR is desensitized and β_2_AR is uncoupled from inotropic Gs/cAMP pathways in failing hearts (Paur et al., 2012). Some beta-blockers are suggested to be effective via the Gi-coupled β_2_AR, once again paradoxically rescuing the heart by making it perform less well (Wisler et al., 2007). In our atrophic model, we see a loss of action/gain of function phenotype which is qualitatively different to hypertrophic or failing hearts. However, we also posit deleterious effects of this pathology on βAR function and a reduction of cardiac functional capacity in atrophic situations as a result of the effects of PMU on membrane structure and organization.

In this study, we employed unloaded cardiomyocytes. Unfortunately, this model system has some limitations. The removal of the cells from their normal configuration in integrated myofibers is somewhat unnatural. It removes them from the normal neurohormonal milieu which they generally experience. Perhaps, more importantly for this study it also removes them from the ‘force’ environment they usually experience. The cells are under cyclic stress and strain throughout the entire life-cycle of the animal and we then study them in a very quiescent state. In some experiments we also employ culture methodology to allow the transduction of FRET reporters it is well known that culture disrupts cellular homeostasis and the TT environment in particular (Pavlović et al., 2010). However, the justification for using isolated cardiomyocytes is that many of the techniques employed within this study are not possible in isolated tissue slices or integrated *ex vivo* preparations. Imaging studies are made complicated by non-specific binding in integrated tissues. Equally, the measurement of cAMP and LTCC activity within cellular microdomains is also currently prohibited in tissue due to the difficulties presented by factors such as the thickness of integrated tissue. Finally, transduction of FRET reporters into isolated cells is also currently a necessity due to practicalities of infecting tissue in, for example, a slice based modality. We also lack transgenic rats expressing FRET sensors, which would be appropriate for this study. A study could be attempted in which the animals could be infected with AAV-9 vectors expressing FRET constructs of interest. This would allow FRET studies to be completed on the day of sacrifice, avoiding cells culture but the necessity for cell isolation would remain.

Mechanical support via ventricular assist devices represents an increasingly common therapy for heart failure. It has functioned as a ‘bridge to transplant’ for many years. Current research is geared toward repurposing mechanical support as a ‘bridge to recovery.’ The latter project requires mechanical unloading to provide curative relief to a heart which is theoretically capable of regenerating its normal function. Bridge to recovery is defined by the number of successful explants of LVADs following the recovery of cardiac function. The rate of explant is around 6% and is usually reliant on heart failure to be in its earliest stages, where minimal cytoarchitectural changes have occurred within the myocytes (Seidel et al., 2017). The exact interplay of the adult hearts lack of intrinsic regenerative capacity and the pathological effects of atrophy is unclear here but must be considered. Indeed, some clinicians are actively pursuing removal of LVADs and some groups are investigating ‘weaning’ protocols (Formica et al., 2010; Selzman et al., 2015). Currently, ‘turn-down’ protocols are employed to assess the heart’s ability to cope on its own with or without adjuvant medical therapy prior to the decision to explant an LVAD. Hemodynamic studies are utilized during ‘turn-down’ to provide this assurance to physicians. The rationale of ‘weaning’ as part of therapy would be to allow the heart to receive enough support to provide sufficient cardiac output and adequate support to regenerate. But this ‘turn-down’ would also appear to offer a training program/stimulus to the damaged myocardium, providing the stimulus to prevent atrophic losses and rebuild essential structures within the cardiomyocytes. In this study, we present data showing the pathological effects of mild withdrawal of load. It would appear that myocardial βAR-cAMP and calcium handling are exquisitely load-sensitive. This should be considered during the provision of mechanical unloading therapy where functional recovery is desired.

## Data Availability Statement

The raw data supporting the conclusions of this manuscript will be made available by the authors, without undue reservation, to any qualified researcher.

## Author Contributions

PW conceived research question, conducted experiments, and authored manuscript. JS-A, CL, CP, and AA-L conducted experiments and performed data analysis. SB conducted experiments. GF, CT, and JG contributed resources and conceived research question.

## Conflict of Interest Statement

The authors declare that the research was conducted in the absence of any commercial or financial relationships that could be construed as a potential conflict of interest.

## Supplementary Material

FIGURE S1 **(A)** Table comparing number of LTCC units vs. number of successful patches (those containing LTCCs). These values are used to calculate the relative occurrence of LTCCs in different cellular microdomains (freshly isolated cells). **(B)** Table comparing the number of LTCC units acquired vs. total number of successful patches: units (patches). These data should be assessed alongside data regarding open probability (freshly isolated cells).

FIGURE S2 **(A)** Example image showing a loaded cardiomyocyte with Cav3 immunostaining (freshly isolated cells). **(B)** Example image showing an unloaded cardiomyocyte with Cav3 immunostaining (freshly isolated cells). **(C)** Graph comparing the density of Cav-3 immunostaining in loaded and unloaded cells (loaded 36.52 ± 1.02% vs. unloaded 33.54 ± 0.77% N,n 3/22-27 mean ± SEM ^∗^*p* < 0.05) (freshly isolated cells). **(D)** Graph comparing the regularity of Cav-3 staining in loaded and unloaded cells (loaded 1.64 × 10^8^± 1.67 × 10^7^ vs. unloaded 0.85 × 10^8^± 1.04 × 10^7^ N,n 3/22-27 mean ± SEM ^∗∗∗^*p* < 0.001) (freshly isolated cells).

FIGURE S3 **(A)** Example image showing a loaded cardiomyocyte stained with di8ANNEPPS (freshly isolated cells). **(B)** Example image showing an unloaded cardiomyocyte stained with di8ANNEPPS (freshly isolated cells). **(C)** Graph comparing the density of di8ANNEPPS staining in loaded and unloaded cardiomyocytes (freshly isolated cells). **(D)** Graph comparing the regularity of di8ANNEPPS staining in loaded and unloaded cardiomyocytes (freshly isolated cells). **(E)** Graph comparing the ratio of cell length vs. width in loaded and unloaded cardiomyocytes (freshly isolated cells). **(F)** Graph comparing the cell area of loaded and unloaded cardiomyocytes (freshly isolated cells).

## Abbreviations

β_(1,2)_AR: beta-adrenergic receptor subtype 1 or 2
BAYK: BAYK8644
cAMP: cyclic adenosine monophosphate
CaMKII: calcium-calmodulin kinase 2
Cav1.2: sub-unit of L-type calcium channel
Cav-3: caveolin-3
cEPAC2: cytosolic epac2 localized cAMP sensor
CGP: CGP20712A
Epac: exchange protein activated by cAMP
FRET: Förster resonance energy transfer
HTHAL: heterotopic heart and lung transplant
ISO: isoprenaline
ICI, ICI118: 551
JPH-2: junctophilin-2
LTCC: L-type calcium channel
LVAD: left ventricular assist device
NKH: NKH477
PKA: protein kinase A
PMU: partial mechanical unloading
P_o_: open probability of LTCC
RII_epac: PKA RII based cAMP sensor
SICM: scanning ion conductance microscopy
TATS: transverse axial tubule system
TT: t-tubule

## References

[B1] Agarwal S. R. MacDougall D. A. Tyser R. Pugh S. D. Calaghan S. C. Harvey R. D. (2011). Effects of cholesterol depletion on compartmentalized cAMP responses in adult cardiac myocytes. *J. Mol. Cell. Cardiol.* 50 500–509. 10.1016/j.yjmcc.2010.11.015 21115018 PMC3049871

[B2] Baker J. G. Hill S. J. Summers R. J. (2011). Evolution of β-blockers: from anti-anginal drugs to ligand-directed signalling. *Trends Pharmacol. Sci.* 32 227–234. 10.1016/j.tips.2011.02.010 21429598 PMC3081074

[B3] Bhargava A. Lin X. Novak P. Mehta K. Korchev Y. Delmar M. (2013). Super-resolution scanning patch clamp reveals clustering of functional ion channels in adult ventricular myocyte. *Circ. Res.* 112 1112–1120. 10.1161/CIRCRESAHA.111.300445 23438901 PMC3899650

[B4] Chen B. Guo A. Zhang C. Chen R. Zhu Y. Hong J. (2013). Critical roles of junctophilin-2 in T-tubule and excitation–contraction coupling maturation during postnatal development. *Cardiovasc. Res.* 100 54–62. 10.1093/cvr/cvt180 23860812 PMC3778961

[B5] Di Benedetto G. Zoccarato A. Lissandron V. Terrin A. Li X. Houslay M. D. (2008). Protein kinase A type I and type II define distinct intracellular signaling compartments. *Circ. Res.* 103 836–844. 10.1161/CIRCRESAHA.108.174813 18757829

[B6] Drakos S. G. Kfoury A. G. Stehlik J. Selzman C. H. Reid B. B. Terrovitis J. V. (2012). Bridge to recovery: understanding the disconnect between clinical and biological outcomes. *Circulation* 126 230–241. 10.1161/CIRCULATIONAHA.111.040261 22777666 PMC3714227

[B7] Engelhardt S. Hein L. Wiesmann F. Lohse M. J. (1999). Progressive hypertrophy and heart failure in beta1-adrenergic receptor transgenic mice. *Proc. Natl. Acad. Sci. U.S.A.* 96 7059–7064. 10.1073/pnas.96.12.7059 10359838 PMC22055

[B8] Formica P. Murthy S. Edwards P. Goldstein D. Maybaum S. (2010). A structured 3-step approach to evaluate cardiac recovery with continuous flow circulatory support. *J. Hear. Lung Transplant.* 29 1440–1442. 10.1016/j.healun.2010.07.008 20817565

[B9] Frisk M. Ruud M. Espe E. K. S. Aronsen J. M. Røe ÅT. Zhang L. (2016). Elevated ventricular wall stress disrupts cardiomyocyte t-tubule structure and calcium homeostasis. *Cardiovasc. Res.* 112 443–451. 10.1093/cvr/cvw111 27226008 PMC5031949

[B10] Holmberg E. Ahn H. Peterzén B. (2017). More than 20 years’ experience of left ventricular assist device implantation at a non-transplant centre. *Scand. Cardiovasc. J.* 51 293–298. 10.1080/14017431.2017.1388536 29029567

[B11] Ibrahim M. Al M. A. Navaratnarajah M. Siedlecka U. Soppa G. K. Moshkov A. (2010). Prolonged mechanical unloading affects cardiomyocyte excitation-contraction coupling, transverse-tubule structure, and the cell surface. *FASEB J.* 24 3321–3329. 10.1096/fj.10-156638 20430793 PMC2923356

[B12] Ibrahim M. Kukadia P. Siedlecka U. Cartledge J. E. Navaratnarajah M. Tokar S. (2012a). Cardiomyocyte Ca^2+^ handling and structure is regulated by degree and duration of mechanical load variation. *J. Cell. Mol. Med.* 16 2910–2918. 10.1111/j.1582-4934.2012.01611.x 22862818 PMC4393719

[B13] Ibrahim M. Navaratnarajah M. Siedlecka U. Rao C. Dias P. Moshkov A. V. (2012b). Mechanical unloading reverses transverse tubule remodelling and normalizes local Ca^2+^-induced Ca^2+^ release in a rodent model of heart failure. *Eur. J. Heart Fail.* 14 571–580. 10.1093/eurjhf/hfs038 22467752 PMC3359860

[B14] Ibrahim M. Navaratnarajah M. Kukadia P. Rao C. Siedlecka U. Cartledge J. E. (2013a). Heterotopic abdominal heart transplantation in rats for functional studies of ventricular unloading. *J. Surg. Res.* 179 31–39. 10.1016/j.jss.2012.01.053 22520576

[B15] Ibrahim M. Navaratnarajah M. Siedlecka U. Rao C. Dias P. Moshkov A. (2013b). Mechanical unloading reverses transverse tubule remodelling and normalises local calcium-induced calcium release in a rodent model of heart failure. *Lancet* 381 S54. 10.1016/S0140-6736(13)60494-8 PMC3359860 22467752

[B16] Lucia C. de Eguchi A. Koch W. J. (2018). New insights in cardiac β-adrenergic signaling during heart failure and aging. *Front. Pharmacol.* 9:904 10.3389/fphar.2018.00904 PMC6095970 30147654

[B17] MacDougall D. A. Agarwal S. R. Stopford E. A. Chu H. Collins J. A. Longster A. L. (2012). Caveolae compartmentalise β2-adrenoceptor signals by curtailing cAMP production and maintaining phosphatase activity in the sarcoplasmic reticulum of the adult ventricular myocyte. *J. Mol. Cell. Cardiol.* 52 388–400. 10.1016/j.yjmcc.2011.06.014 21740911 PMC3270222

[B18] Nguyen M.-N. Kiriazis H. Ruggiero D. Gao X.-M. Su Y. Jian A. (2015). Spontaneous ventricular tachyarrhythmias in β2-adrenoceptor transgenic mice in relation to cardiac interstitial fibrosis. *Am. J. Physiol. Circ. Physiol.* 309 H946–H957. 10.1152/ajpheart.00405.2015 26116714

[B19] Nikolaev V. O. Bünemann M. Hein L. Hannawacker A. Lohse M. J. (2004). Novel single chain cAMP sensors for receptor-induced signal propagation. *J. Biol. Chem.* 279 37215–37218. 10.1074/jbc.C400302200 15231839

[B20] Nikolaev V. O. Moshkov A. Lyon A. R. Miragoli M. Novak P. Paur H. (2010). β2-adrenergic receptor redistribution in heart failure changes cAMP compartmentation. *Science* 327 1653–1657. 10.1126/science.1185988 20185685

[B21] Novak P. Li C. Shevchuk A. I. Stepanyan R. Caldwell M. Hughes S. (2009). Nanoscale live-cell imaging using hopping probe ion conductance microscopy. *Nat. Methods* 6 279–281. 10.1038/nmeth.1306 19252505 PMC2702483

[B22] Paur H. Wright P. T. Sikkel M. B. Tranter M. H. Mansfield C. O’Gara P. (2012). high levels of circulating epinephrine trigger apical cardiodepression in a β(2)-Adrenoceptor/Gi-dependent manner: a new model of takotsubo cardiomyopathy. *Circulation* 126 697–706. 10.1161/CIRCULATIONAHA.112.111591 22732314 PMC4890655

[B23] Pavlović D. McLatchie L. M. Shattock M. J. (2010). The rate of loss of T-tubules in cultured adult ventricular myocytes is species dependent. *Exp. Physiol.* 95 518–527. 10.1113/expphysiol.2009.052126 20061354

[B24] Sanchez-Alonso J. L. Bhargava A. O’Hara T. Glukhov A. V. Schobesberger S. Bhogal N. (2016). Microdomain-specific modulation of l-type calcium channels leads to triggered ventricular arrhythmia in heart failure. *Circ. Res.* 119 944–955. 10.1161/CIRCRESAHA.116.308698 27572487 PMC5045818

[B25] Schobesberger S. Wright P. Tokar S. Bhargava A. Mansfield C. Glukhov A. V. (2017). T-tubule remodelling disturbs localized β2-adrenergic signalling in rat ventricular myocytes during the progression of heart failure. *Cardiovasc. Res.* 113 770–782. 10.1093/cvr/cvx074 28505272 PMC5437368

[B26] Seidel T. Navankasattusas S. Ahmad A. Diakos N. A. Xu W. D. Tristani-Firouzi M. (2017). Sheet-like remodeling of the transverse tubular system in human heart failure impairs excitation-contraction coupling and functional recovery by mechanical unloading. *Circulation* 135 1632–1645. 10.1161/CIRCULATIONAHA.116.024470 28073805 PMC5404964

[B27] Selzman C. H. Madden J. L. Healy A. H. McKellar S. H. Koliopoulou A. Stehlik J. (2015). Bridge to removal: a paradigm shift for left ventricular assist device therapy. *Ann. Thorac. Surg.* 99 360–367. 10.1016/j.athoracsur.2014.07.061 25442985 PMC4283551

[B28] Terracciano C. Al-Masri A. Siedlecka U. Soppa G. K. Navaratnarajah M. Hadjiphilippou S. (2009). Chronic mechanical unloading of rat hearts disrupts local calcium-induced calcium release in isolated cardiomyocytes. *Eur. Heart J.* 30:176.

[B29] Watson S. A. Perbellini F. Terracciano C. M. (2016). Cardiac t-tubules: where structural plasticity meets functional adaptation. *Cardiovasc. Res.* 112 423–425. 10.1093/cvr/cvw198 27659500

[B30] Wisler J. W. DeWire S. M. Whalen E. J. Violin J. D. Drake M. T. Ahn S. (2007). A unique mechanism of β-blocker action: carvedilol stimulates β-arrestin signaling. *Proc. Natl. Acad. Sci. U.S.A.* 104 16657–16662. 10.1073/pnas.0707936104 17925438 PMC2034221

[B31] Wong J. Baddeley D. Bushong E. A. Yu Z. Ellisman M. H. Hoshijima M. (2013). Nanoscale distribution of ryanodine receptors and caveolin-3 in mouse ventricular myocytes: dilation of t-tubules near junctions. *Biophys. J.* 104 L22–L24. 10.1016/j.bpj.2013.02.059 23746531 PMC3672889

[B32] Wright P. T. Bhogal N. K. Diakonov I. Pannell L. M. K. Perera R. K. Bork N. I. (2018). Cardiomyocyte membrane structure and cAMP compartmentation produce anatomical variation in β2AR-cAMP responsiveness in murine hearts. *Cell Rep.* 23 459–469. 10.1016/j.celrep.2018.03.053 29642004 PMC5912947

[B33] Wright P. T. Nikolaev V. O. O’Hara T. Diakonov I. Bhargava A. Tokar S. (2014). Caveolin-3 regulates compartmentation of cardiomyocyte beta2-adrenergic receptor-mediated cAMP signaling. *J. Mol. Cell. Cardiol.* 67 38–48. 10.1016/j.yjmcc.2013.12.003 24345421 PMC4266930

