# Supplementary figures and images for: Partial Mechanical Unloading of the Heart Disrupts L-Type Calcium Channel and Beta-Adrenoceptor Signaling Microdomains
Source: Front Physiol. 2018 Sep 19;9:1302. doi: 10.3389/fphys.2018.01302 (PMC6157487; doi:10.3389/fphys.2018.01302)

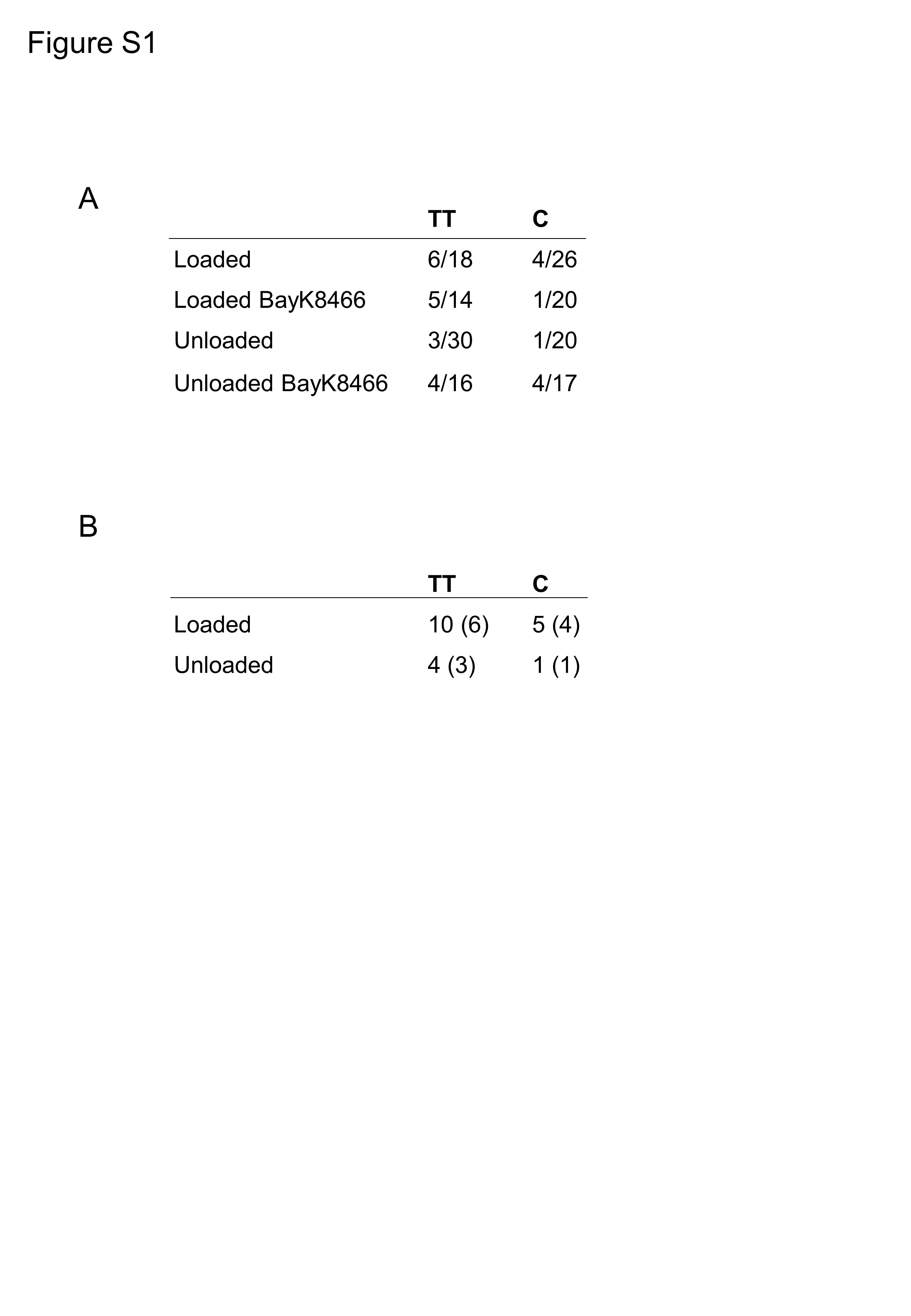

Supplement: FIGURE S1 — (A) Table comparing number of LTCC units vs. number of successful patches (those containing LTCCs). These values are used to calculate the relative occurrence of LTCCs in different cellular microdomains (freshly isolated cells). (B) Table comparing the number of LTCC units acquired vs. total number of successful patches: units (patches). These data should be assessed alongside data regarding open probability (freshly isolated cells). [file Image_1.tif]

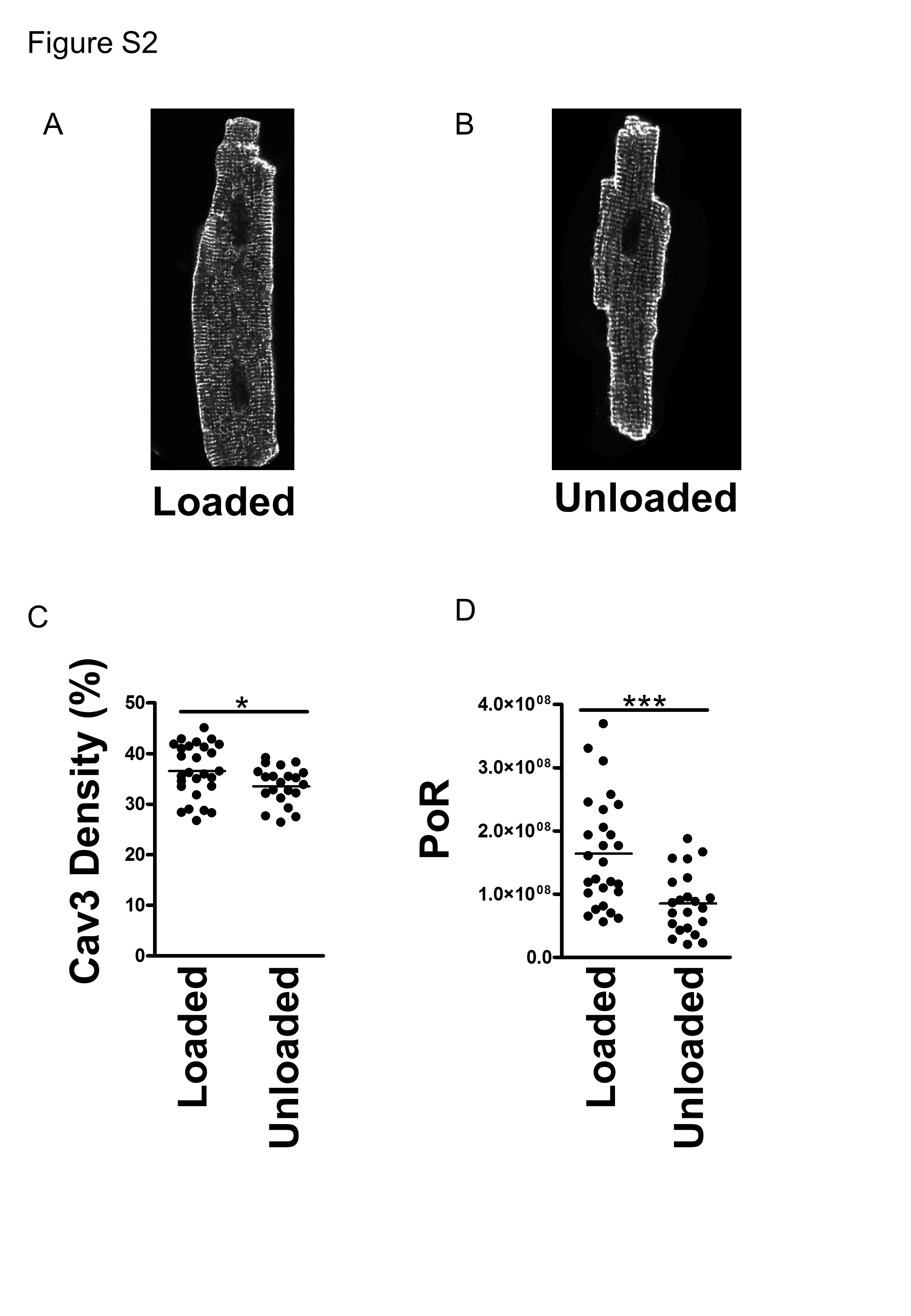

Supplement: FIGURE S2 — (A) Example image showing a loaded cardiomyocyte with Cav3 immunostaining (freshly isolated cells). (B) Example image showing an unloaded cardiomyocyte with Cav3 immunostaining (freshly isolated cells). (C) Graph comparing the density of Cav-3 immunostaining in loaded and unloaded cells (loaded 36.52 ± 1.02% vs. unloaded 33.54 ± 0.77% N,n 3/22-27 mean ± SEM ∗p < 0.05) (freshly isolated cells). (D) Graph comparing the regularity of Cav-3 staining in loaded and unloaded cells (loaded 1.64 × 10^8^± 1.67 × 10^7^ vs. unloaded 0.85 × 10^8^± 1.04 × 10^7^ N,n 3/22-27 mean ± SEM ∗∗∗p < 0.001) (freshly isolated cells). [file Image_2.tif]

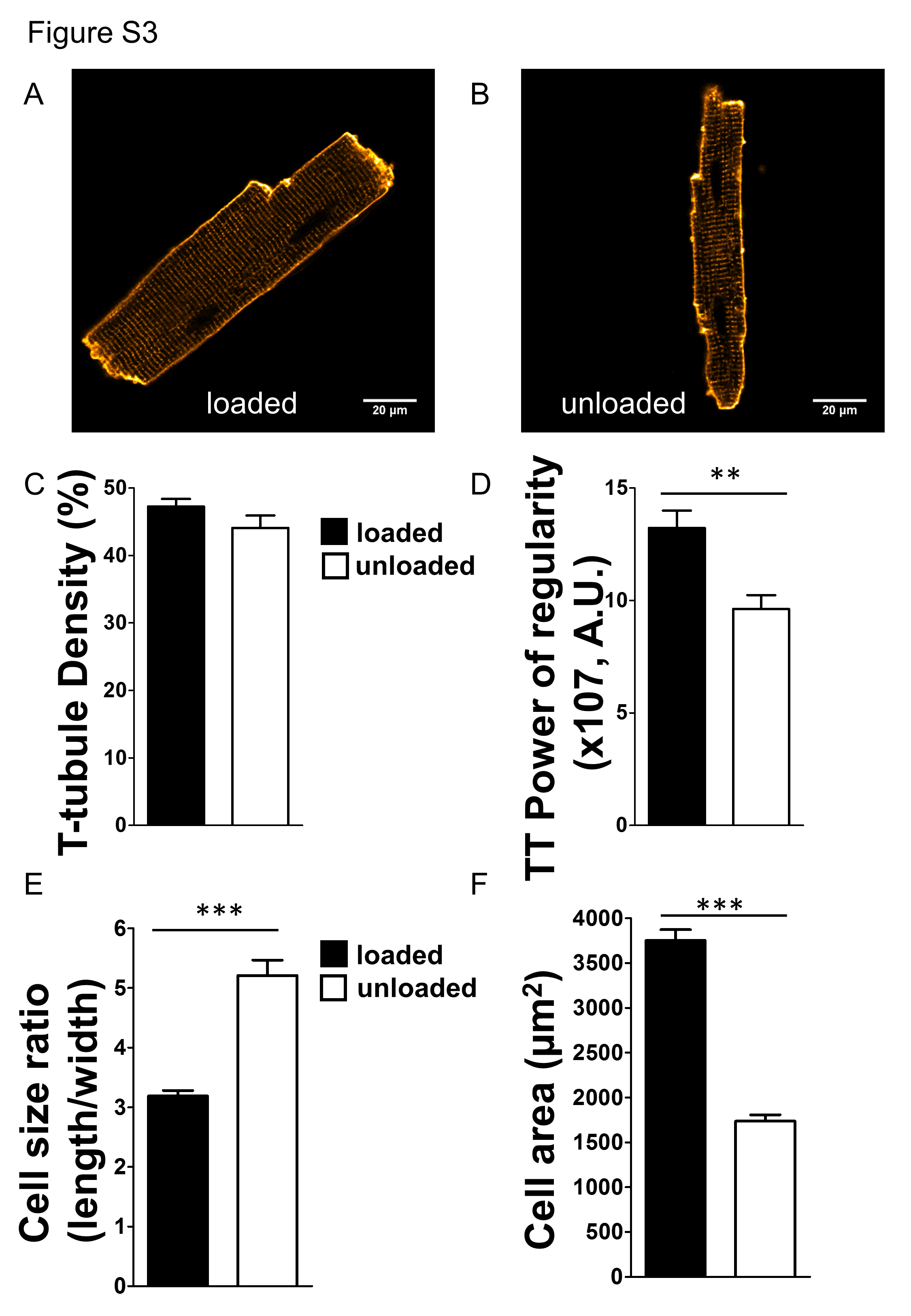

Supplement: FIGURE S3 — (A) Example image showing a loaded cardiomyocyte stained with di8ANNEPPS (freshly isolated cells). (B) Example image showing an unloaded cardiomyocyte stained with di8ANNEPPS (freshly isolated cells). (C) Graph comparing the density of di8ANNEPPS staining in loaded and unloaded cardiomyocytes (freshly isolated cells). (D) Graph comparing the regularity of di8ANNEPPS staining in loaded and unloaded cardiomyocytes (freshly isolated cells). (E) Graph comparing the ratio of cell length vs. width in loaded and unloaded cardiomyocytes (freshly isolated cells). (F) Graph comparing the cell area of loaded and unloaded cardiomyocytes (freshly isolated cells). [file Image_3.tif]
